# *Mangifera indica* L. Leaf Extract Induces Adiponectin and Regulates Adipogenesis

**DOI:** 10.3390/ijms20133211

**Published:** 2019-06-29

**Authors:** Giuseppe Sferrazzo, Rosa Palmeri, Luca Vanella, Lucia Parafati, Simone Ronsisvalle, Antonio Biondi, Francesco Basile, Giovanni Li Volti, Ignazio Barbagallo

**Affiliations:** 1 Department of Drug Science, Biochemistry Section, University of Catania, Viale A. Doria 6, 95125 Catania, Italy; 2 Department of Agricultural, Food and Environment, University of Catania, Via S. Sofia, 95125 Catania, Italy; 3 Department of General Surgery and Medical-Surgical Specialties, University of Catania, Via S. Sofia 87, 95125 Catania, Italy; 4 Department of Biomedical and Biotechnological Sciences, University of Catania, Via S. Sofia 87, 95125 Catania, Italy; 5 EuroMediterranean Institute of Science and Technology, Via Michele Miraglia 20, 90139 Palermo, Italy

**Keywords:** oxidative stress, metabolic syndrome, adipocyte, adiponectin, mangiferin

## Abstract

Natural bioactive compounds may be used in obese patients because of their ability to impact on various key mechanisms involved in the complex pathophysiological mechanisms of such condition. The aim of this study was to investigate the effect of a *Mangifera indica* L. leaf extract (MLE) on adipogenic differentiation of murine preadipocyte cells. 3T3-L1 cells were treated during their differentiation with various concentrations of (*Mangifera indica* L.) leaves extract (MLE) (750, 380, 150, 75 and 35 μg) in order to assess their lipid content, adiponectin production, expression profile of genes involved in lipid metabolism, oxidative stress and inflammation. Our results showed that MLE was particularly enriched in polyphenols (46.30 ± 0.083 mg/g) and that pharmacological treatment of cells resulted in a significant increase of adiponectin levels and reduction of intracellular lipid content. Consistently with these results, MLE resulted in a significant decrease of the expression of genes involved in lipid metabolism (FAS, PPARG, DGAT1, DGAT2, and SCD-1). In conclusion, our results suggest that MLE may represent a possible pharmacological tool for obese or metabolic syndrome patients.

## 1. Introduction

Currently, there is a growing interest in finding new bioactive compounds of plants and fruits that could be exploited for their potential role of counteracting cardiovascular diseases and certain types of cancer [1]. Bioactive compounds are secondary metabolites synthesized from plants in response to stress conditions [2]. To this regard, several studies focused on beneficial effects of fruits and vegetables, including their phytochemical content and bioavailability, in order to determine the effects on health/disease risk endpoints [3,4,5].

*Mangifera indica* L., commonly known as mango, is a fruit belonging to the family of *Anacardiaceae* in the order of *Sapindales* [6]. This fruit, typical of tropical and sub-tropical regions, is one of the most popular edible fruits and its production ranks seventh in world fruit production [7]. Mango is considered a good source of antioxidants and water- and lipid-soluble micronutrients, such as ascorbic acid, carbohydrates, roughage, carotenoids, organic acids and phenolic compounds [8]. The main phenolic and other polar compounds identified in mango are flavonol glycosides, xanthone glycosides, gallotannins, benzophenones and anthocyanins [9]. Mango extracts obtained by different plant and fruit materials are worldwide used in traditional medicine, mainly for treating diarrhea, kidney and urinary disorders, as complementary therapy to manage type 2 diabetes, against inflammation, oxidative stress [10], cancer [11] and obesity [12].

Obesity is a serious worldwide health problem with a complex multifactorial etiology causing a high incidence of mortality and morbidity.

Adipose tissue may be considered an endocrine organ since it produces and secretes multiple immune-modulatory proteins known as adipokines [13]. These cytokines can be classified into proinflammatory adipokines (i.e., IL-6), and anti-inflammatory adipokines (i.e., adiponectin). In obesity there exists an imbalance of adipocytes function characterized by an increased expression of pro-inflammatory adipokines and diminished expression of anti-inflammatory adipokines leading to a chronic, low-grade inflammatory state [14] and promoting metabolic dysfunction and cardiovascular disease. Anti-inflammatory adipokines, such as adiponectin are preferentially produced by non-obese patients, but its expression is downregulated in the dysfunctional adipocytes of obese patients [15]. Consistently, previous studies suggested that adiponectin expression is inhibited in adipocytes by pro-inflammatory cytokines, hypoxia, and oxidative stress [16,17].

The aim of this study was to investigate the effect of a *Mangifera indica* L. leaf extract (MLE) on adipogenic differentiation of murine preadipocyte 3T3-L1 cells.

## 2. Results

### 2.1. Polyphenols Determination

The polyphenols concentration in the leaves extract was very high compared to other plants with a value of 46.30 ± 0.083 mg/g.

### 2.2. UPLC-MS/MS

Leaf derived samples were lyophilized and analyzed by UPLC/Ms-Ms techniques. Quantitative analysis was obtained using C18 Phenomenex Luna column. Mangiferin, myo-inositol and γ-Orizanolo were characterized in leaf samples and standards were acquired and utilized for calibration line. The results of the quantitative analysis are reported in Table 1. The detected amount of mangiferin resulted higher than limit of quantification (LOQ) in all samples equal to 70,200 ng/mL with 2.36 of retention time. Interestingly, concentrations reported for γ-orizanolo (47,700 ng/mL; retention time 2.15 min) and myo-inositol (21,600 ng/mL; rt. 2.17 min) were particularly relevant. These results are of good biological and clinical interest in the light of multiple positive biological activities of described compounds.

### 2.3. Radical Scavenging Activity of MLE

**Figure 1:**
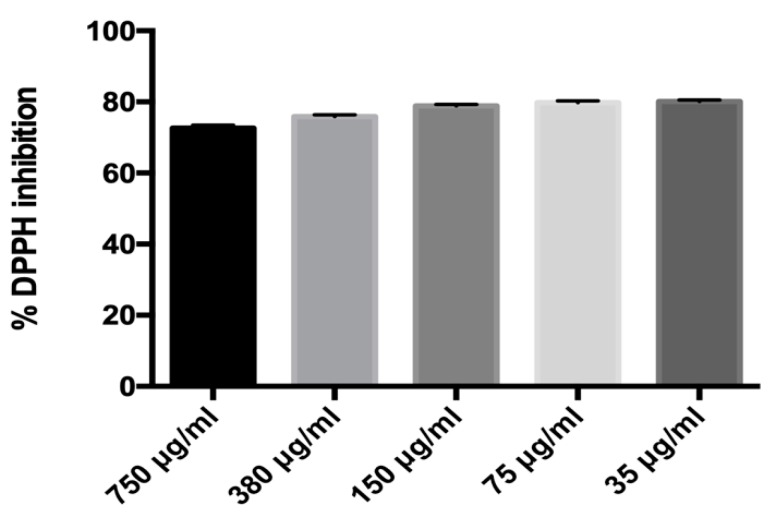
In vitro antioxidant activity measured through percentage of DPPH Inhibition.

MLE antioxidant activity was evaluated through the DPPH test. Figure 1 shows the effect of the ethanol extract at different concentrations; 750 μg, 380 μg, 150 μg, 75 μg and 35 μg. Our data demonstrated that all concentrations tested resulted in about 80% of DPPH inhibition.

### 2.4. Effect of MLE on Cell Viability

**Figure 2:**
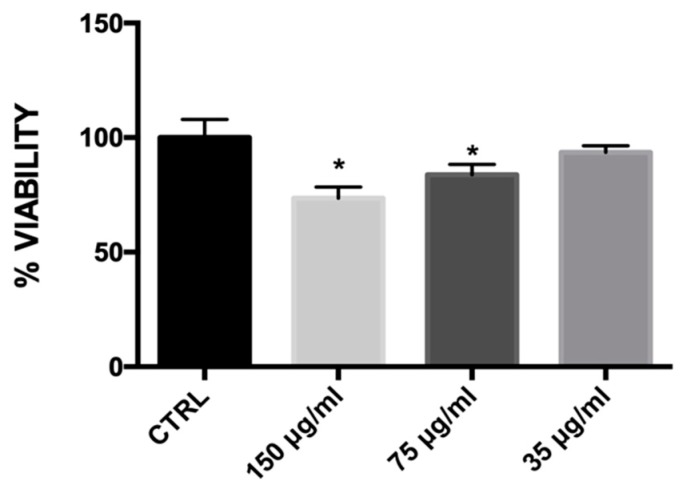
The viability assay in 3T3-L1 cells measured by bromide 3-(4,5-dimethylthiazol-2-yl)-2,5-diphenyltetrazolium (MTT) assay. Bars represent the mean ± SEM of six independent experiments. * *p* < 0.05 versus control (CTRL) cells.

Figure 2 shows that tested concentrations, 150 μg and 75 μg exhibited only marginal cytotoxicity. Contrarily, no significant differences were observed in the cells treated with 35 μg of MLE compared with the control group indicating a negligible in vitro cytotoxic activity. Accordingly, only the concentration of 35 μg/mL was used for successive experiments.

### 2.5. Inhibition of α-Glucosidase

**Figure 3:**
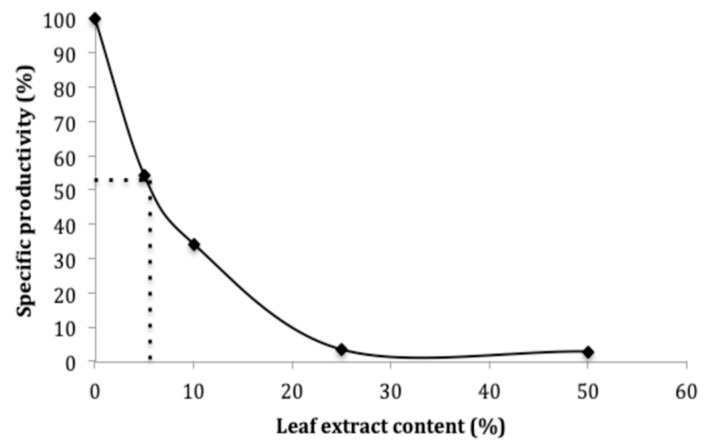
Inhibition of α-glucosidase through MLE. The IC50 values were graphically determined as the half-maximal inhibitory concentration of the inhibitor species giving 50% inhibition. All assays were performed in triplicate.

The inhibition of α-glucosidase activity from *S. cerevisiae* (E.C. 3.2.1.20) following MLE exposure was examined. Figure 3 shows that the percentage of α-glucosidase inhibition increased along with leaf extract concentration in a dose dependent manner. The extract showed 50% of enzyme inhibition (IC50) at a concentration of 7%. The low IC50 value for mango leaf extract may be due to the presence of phenolic acids, flavonoids, mangiferin and their derivatives found in the extract.

### 2.6. Analysis of Adipogenic Differentiation in 3T3-L1 Cells

**Figure 4:**
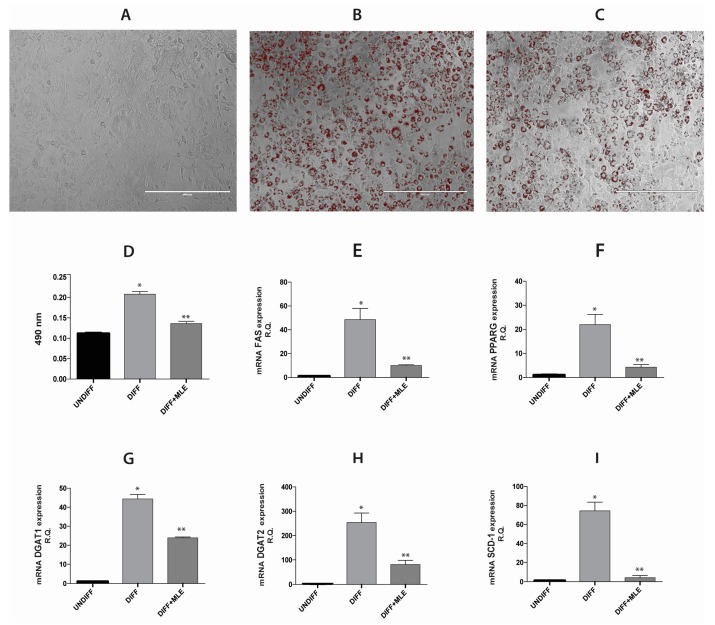
Analysis of adipogenic differentiation in 3T3-L1 cells. Images (**A**–C) microscopy are of cells after differentiation. Oil red staining in undifferentiated cells (**A**), differentiated cells (**B**) and differentiated + MLE (**C**). In Figure (**D**); quantitative oil red staining measured by spectrophotometer at 490 nm reading. Images (**E**–**I**), show a quantitative analysis of gene expression of FAS (**E**), PPARγ (**F**), DGAT1 (**G**), DGAT2 (**H**) and SCD1 (**I**) after adipogenic differentiation. Bars represent the mean ± SD of six independent experiments. * *p* < 0.05 versus undifferentiated cells; ** *p* < 0.05 versus differentiated cells.

3T3-L1 pre-adipocytes were differentiated for five days with or without of MLE (35 μg/mL). To determine lipid content, after five days of treatment, the cells were fixed and stained with oil red. Undifferentiated cells (Figure 4A), grown with DMEM 4.5 g/L d-glucose, did not exhibit lipid accumulation. Differentiated cells (Figure 4B) accumulated lipids at day five, whereas cells grown with adipogenic medium and treated with MLE (35 μg/mL) (Figure 4C) showed a significantly inhibition of lipid accumulation. Figure 4D shows a significant and negative association between the MLE effect and differentiation of 3T3-L1 pre-adipocytes into adipocyte measured by spectrophotometer at 490 nm.

Consistent with the results obtained by the oil red staining, in 3T3-L1 cells we evaluated the expression of the following adipogenic markers: Peroxisome proliferator activated receptor-gamma (PPAR-γ), fatty acid synthase (FAS), diacylglycerol-o-acyltransferase-1 (DGAT-1), diacylglycerol-o-acyltransferase-2 (DGAT-2) and stearoyl-coenzyme A desaturase 1 (SCD-1). The data (Figure 4E–I) showed that their expression was upregulated in differentiated group, while in the third group (differentiated + MLE) it was downregulated. These results suggest that MLE treatment reduces the adipogenic differentiation of pre-adipocytes.

### 2.7. MLE Restores Glutathione Resources after Differentiation

**Figure 5:**
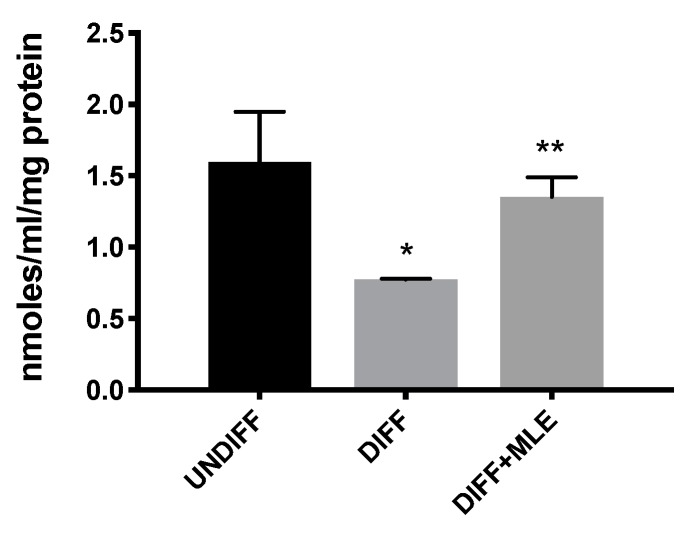
Glutathione cellular content after adipogenic differentiation. Bars represent the mean ± SEM of six independent experiments. * *p* < 0.05 versus undifferentiated cells; ** *p* < 0.05 versus differentiated cells.

To further evaluate the possible involvement of adipogenic differentiation and the MLE effect on glutathione, we measured its content by enzymatic activity. We observed a significantly depletion of glutathione (GSH) after differentiation (Figure 5) while MLE treatment was able to restore glutathione cellular resources.

### 2.8. MLE Effects on Oxidative Stress and Inflammatory Markers Expression

**Figure 6:**
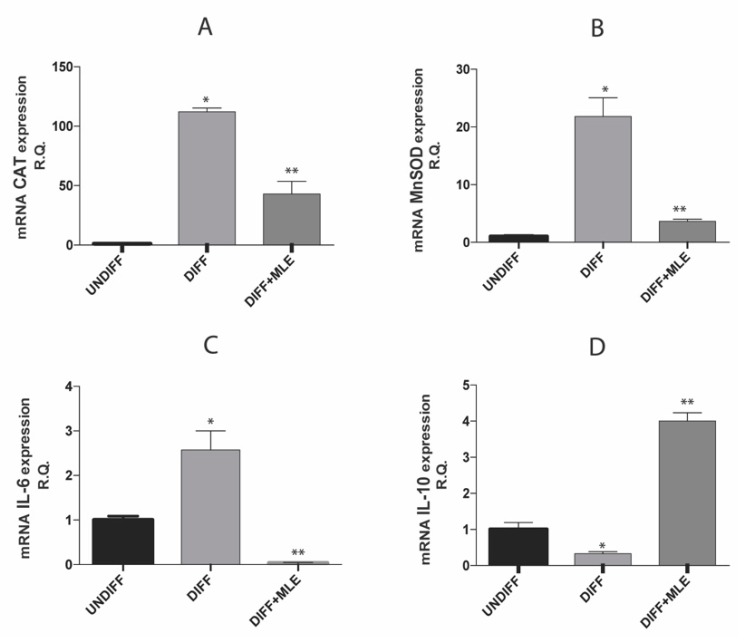
Gene expression CAT (**A**), MnSOD (**B**), IL-6 (**C**) and IL-10 (**D**) evaluated by RT-PCR. Bars represent the mean ± SEM of six independent experiments. * *p* < 0.05 versus undifferentiated cells; ** *p* < 0.05 versus differentiated cells.

In order to evaluate the antioxidant and anti-inflammatory effects of MLE, we evaluated the gene expression of catalase (CAT), manganese-dependent superoxide dismutase (MnSOD), heme oxygenase-1 (HO-1), IL-6 and interleukin-10 (IL-10). Figure 6 shows that in differentiated cells gene expression of CAT (A), MnSOD (B)- and pro-inflammatory cytokine IL-6 (C) resulted overexpressed, while co-treatment with MLE was able to decrease expression of all these genes. In accordance with these results, we found that MLE treatment significantly increased anti-inflammatory gene IL-10 (D) compared to the others treatment groups. These results suggest that treatment with MLE reduces inflammation and oxidative stress levels in differentiated cells.

### 2.9. MLE Effect on PPAR-α, Heme oxygenase 1 (HO-1) and Adiponectin Expression

**Figure 7:**
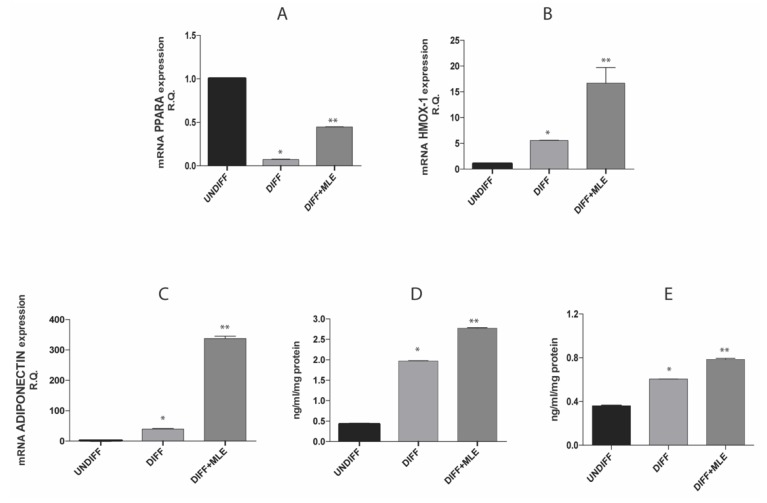
Gene expression of PPAR-α (**A**), HO-1 (**B**) and adiponectin (**C**) evaluated by RT-PCR. In image (**D**) protein adiponectin cellular levels and in (**E**) protein adiponectin released in cell culture supernatant tested by enzyme-linked immunosorbent assay (ELISA). Bars represent the mean ± SEM of six independent experiments. * *p* < 0.05 versus undifferentiated cells; ** *p* < 0.05 versus differentiated cells.

In order to investigate a possible biochemical pathway underlying MLE beneficial effects, we assessed the expression levels of peroxisome proliferator activated receptor-alpha (PPAR-α), HO-1 and adiponectin. Figure 7 shows that expression level of PPAR-α (Figure 7A) was reduced in differentiated group while an opposite result was obtained in MLE group. HO-1 and adiponectin gene expression increased after differentiation (Figure 7B,C). Moreover, MLE treatment during differentiation was able to overexpress both gene levels compared to differentiated cells (Figure 7B,C). In addition, adiponectin protein level was significantly (*p* < 0.05) higher in 3T3-L1 cell treated with MLE compared to differentiated group (Figure 7D). Likewise, the greater amount of adiponectin released in cell culture supernatant was found in the group treated with MLE (Figure 7E).

## 3. Discussion

Several studies suggest that extracts of various part of plant *Mangifera indica* L. show a protective effect on different human pathologies [6,19]. In particular, this extract acts as scavenger of free radical and consequently may exhibit beneficial pharmacological effects in several pathologies in which oxidative stress [20,21] plays a major role. In addition, plants cultivated under abiotic stress condition express various secondary metabolite such us polyphenol, alkaloids and terpenes [22,23].

Among these, mangiferin has been investigated for its many beneficial effects on inflammation, infections and pain. Mangiferin shows many beneficial biological activities, such as anti-inflammatory, antioxidant, hypolipidemic, and antihyperglycemic effects [24,25]. Recent studies found that mangiferin decreased serum triglycerides (TG) and free fatty acids (FFA) levels in hyperlipidemic hamsters and rats by inhibiting lipogenesis and promoting fatty acid oxidation [26]. Furthermore, some studies showed that mangiferin may improve insulin resistance both in vivo and in vitro [27].

In our study, we used the leaves of *Mangifera indica* grown in Sicily (Italy), where the mild climate is characterized by constant changes in temperature, water and humidity conferring special properties to the plants.

Our data showed that MLE possessed a high concentration of total polyphenols and phytochemical compounds; in particular, among all of them, we found a high content of mangiferin, myo-inositol and γ-oryzanol (Orz). Myo-inositol is a stereoisomer of inositol, a polyol belongs to vitamin B, that is currently used for the treatment of polycystic ovary syndrome [28]. Previous studies showed that myo-inositol regulates glucose metabolism and transport [29], moreover, Shorkpour et al., showed that Myo-inositol supplementation in women with polycystic ovary syndrome, compared with metformin, significantly reduced fasting plasma glucose, serum insulin levels, homeostasis model of assessment-insulin resistance, serum triglycerides, and significantly increased the quantitative insulin sensitivity check index compared with metformin [30]. 

Orz is a ferulic acid ester compound of several kinds of triterpene alcohol and phytosterol [31]. A recent study reported that Orz decreases high fat diet induced endoplasmic reticulum stress in pancreatic β-cells improving β-cell function directly acting on pancreatic islets and enhances glucose-stimulated insulin secretion [31]. Consistent with these observations, our results showed an in vitro inhibition of α-glucosidase, a glycoside hydrolase which hydrolyses the terminal, non-reducing-1→4-linked α-d-glucose residues and then releases glucose into blood resulting in postprandial hyperglycemia [32].

Dysfunctional adipose tissue lipid metabolism leads to increased circulating free fatty acids, initiating oxidative stress and inflammatory signaling cascades. Therefore, we analyzed the possible effect of MLE on adipogenic differentiation of 3T3 preadipocytes cells. Our results suggested that MLE treatment was able to decrease lipid accumulation and adipogenic differentiation markers. Moreover, we showed that adipogenic differentiation under hyperglicemia condition decreased GSH cellular resource and increased inflammatory and oxidative stress markers such as IL-6, MnSOD, CAT and HO-1. These results are consistent with previous studies reporting that decreased cellular GSH content is associated with impaired insulin response, raising the possibility that decreased GSH is causative in impairing insulin action. Moreover, it was demonstrated that 3T3-L1 adipocytes exposed to oxidative stress were characterized by decreased GSH and impaired in insulin-stimulated glucose transport and glucose transporters GLUT4 (glucose transporter 4) translocation [33]. In our experiment we showed that MLE restored GSH content during adipogenesis and decreased anti-inflammatory and antioxidant genes. In addition, MLE treatment during adipogenic differentiation was also able to increase the gene levels of anti-inflammatory cytokine IL-10. Interestingly, IL-10 in obese mice, displayed significant anti-obesity and anti-inflammatory effects, such as reduced serum total cholesterol, adipocyte size, proinflammatory cytokine secretion IL-6 in adipose tissue [34].

In order to investigate the biochemistry mechanism, we analyzed the gene levels of HO-1, PPAR-α and adiponectin expression. Previous reports suggested that PPAR-α activation prevents inflammation in adipose tissue and enhances the action of adiponectin in the amelioration of obesity-induced insulin resistance [35]. Adiponectin is the most abundant peptide hormone secreted by adipocytes which performs many metabolic functions that link to energy metabolism [36]. It mediates insulin sensitivity in skeletal muscle through AMPK (adenosine monophosphate-activated protein kinase) and peroxisome proliferator-activated receptor alpha (PPAR-α). Adiponectin enhances basal glucose and insulin-stimulated glucose uptake in adipose tissues via AMPK activation and its levels demonstrated an inverse correlation with adiposity and proinflammatory cytokines in patients suffering from metabolic syndrome [37,38]. Furthermore, several experimental models indicated that adiponectin protects against obesity-linked metabolic disease. The acute administration of adiponectin leads to an improvement in metabolic parameters in a mouse model of obesity [17]. Conversely, adiponectin-deficient mice develop greater insulin resistance when placed on a high calorie diet [39,40], whereas the transgenic overexpression of adiponectin in ob/ob mice improves metabolic parameters independently of weight loss [41]. Moreover, it has recently reported a beneficial effect of heme oxygenase-1 (HO-1)/adiponectin axis on cardiovascular disease [42,43]. HO-1 is the major enzyme of heme metabolism decomposition and can be induced by various oxidative insults [44]. Upregulation of HO-1 may induce an increase in adiponectin secretion by remodeling adipose tissue, which prevents endothelial dysfunction by its anti-oxidative and anti-inflammatory effects [45,46,47,48]. 

In this study, we reported that MLE treatment during adipogenic differentiation of preadipocytes increased the expression of HO-1 and PPAR-α, cellular adiponectin expression and extracellular adiponectin secretion, confirming that phytochemical compound contained in *Mangifera indica* leaves possess beneficial activity on adipocytes, ameliorating lipid metabolism and functionality.

## 4. Materials and Methods

### 4.1. Leaves Extract Preparation

Samples of mango (*Mangifera indica* L.) leaves obtained from the cultivar “Kensington pride” grown in Sicily (Italy) were used. The leaves were collected at the same time, during pruning period, from Sicilian organic farming. Leaves were dried in a stove at 36 °C (constant temperature). The extract was obtained accordingly to the Yi Zhang et al. (2013) [49] method, with some adjustments. Briefly, the natural extract preparation had been performed through the crushing of plant material using pestle and mortar until a homogeneous sample was obtained. Then, the sample was extracted in 70% ethanol (*v*/*v*) for 30 min. Subsequently, the sample was filtered by a vacuum pump and stored in the dark at –20 °C.

### 4.2. Total Polyphenols Content

Total polyphenol content of MLE was evaluated by the Folin–Ciocalteu method [50], with some adjustments. Briefly, 1.25 mL portion of Folin–Ciocalteu (Fluka, Milan, Italy) reagent was mixed with 0.25 mL of the sample; after 3 min, 2.5 mL of a sodium carbonate solution (20%) was added to the mixture and the reaction was conducted in the dark for 1 h. The absorbance was spectrophotometrically measured at 725 nm, using a Perkin elmer lambda 25 uv-vis spectrophotometer. Gallic acid (Fluka, Milan, Italy) was used as standard for calibration curve (0.02–0.8 mg/mL; *y* = 1.1429*x* + 0.0185, where *x* and *y* represent the caffeic acid concentration (mg/mL) and absorbance at 725 nm, respectively; *r*^2^ = 0.9995). Contents of total phenolic compounds in MLE were expressed as gallic acid equivalents in milligram per gram of dried leaves [50].

### 4.3. UPLC-Ms/Ms Analisys

An analytical method based on ultra-performance liquid chromatography coupled to mass spectrometry UPLC-Ms/Ms (AB SCIEX API 2000TM/Perkin-Elmer Flexar FX-10, Milan, Italy) was used to obtain a separation of phytochemical compounds. The better separation was achieved using 0.1% acetic acid and acetonitrile as mobile phase (30:70 *v*/*v* at 500 µL/min) utilizing a C18 column (Phenomenex Luna, Bologna, Italy, 5 µm, 15 × 0.1 cm). The following elution program was carried out: From 0 to 10 min at 30:70 *v*/*v*. A total volume of 20 µL was injected. The UV detector was monitored to 220 nm.

ESI-Ms/Ms was used in positive and negative polarities for a comprehensive assessment of the analyzed spectrum and the positive one was selected for detection of compounds. Other relevant settings: Curtain gas 30, Ions pray Voltage (IS) 5500, Ion source gas1 (GS1) 60, Ion source gas2 (GS2) 30 and interface heater (ON), delustering potential (DP) 163.53, focusing potential (FP) 400.0, and entrance potential (EP) 6.86. Temperature (TEM) 450 °C. The mangiferin was also evaluated by biphenyl column (Phenomenex Kinetex, Bologna, Italy, 2.6 μm Biphenyl 100A 100 × 2.1 mm). The mass in full scan was accomplished in a range which covered from 50 to 1000 Da; the atomizing gas was nitrogen. Mangiferin, myo-inositol and γ-orizanol were recognized by the molecular weight (423.02, 181.58 and 603.40 [M + H]^+^), the retention time and their fragmentation were compared with data of chromatography and mass standards. Each product was analyzed by analysis Q1/Q3 fragmentation and specific mass scanning. [18] Data acquisition, peak integration, and calibrations were performed with Analyst^®^ Software (AB Sciex, Milan, Italy).

The stock solutions were prepared using 1 mg in 10 mL of LC/Ms grade methanol. Six dilutions were made for each standard with the following final concentrations: 0.017 mg/mL, 0.0028 mg/mL, 0.000463 mg/mL, 7.72 × 10^−5^ mg/mL, 1.286 × 10^−5^ mg/mL and 2.143 × 10^−6^ mg/mL. All the solutions were kept at −20 °C in the dark, for the duration of the preparation of calibration. Linear regression was used for the calibration of standard line on which were placed the standard compounds.

### 4.4. Antioxidant Capacity

Radical scavenging activity was determined on MLE by the DPPH, as previously described. Particularly, an ethanol 1,1-Diphenyl-2-picryl-hydrazyl DPPH• (Sigma–Aldrich, St. Louis, MI, USA) solution (100 μM) was used and 5.7 μL of solution was mixed with 194.3 μL of leaf extract. The samples were incubated for 20 min in the dark and at room temperature, then the loss of absorbance at 515 nm (AE) was measured spectrophotometrically. DPPH radicals have a maximum absorption at 515 nm, the peak disappears with reduction by an antioxidant compound. A blank sample containing 200 μL of ethanol was used as reference. The experiment was carried out in triplicate. In addition, radical scavenging activity (RSA%) was estimated using the following equation:

RSA% = [(AB − AE)/AB] × 100(1)

AB; absorbance of the blank sample, and AE; absorbance of the leaf extract. (Data represent the percentage of DPPH radical inhibition).

### 4.5. Measurement of Cell Viability: Bromide 3-(4,5-dimethylthiazol-2-yl)-2,5-diphenyltetrazolium (MTT) Assay

Cell cultures were treated for 24 h in 96-well plates with different concentrations of MLE, respectively 150 μg, 75 μg and 35 μg. Successively, the medium was replaced by a solution containing bromide 3-(4,5-dimethylthiazol-2-yl)-2,5-diphenyltetrazolium (MTT) and incubated for 3 h at 37 °C. Finally, every plate well was added with 100 μL of dimethyl sulfoxide (DMSO) and then read by a spectrophotometer at *λ* = 570 nm.

### 4.6. Alpha-Glucosidase Assay

An α-glucosidase from *S. cerevisiae* (E.C. 3.2.1.20) was prepared in potassium phosphate (0.1 mol/L, 3.2 mmol/L MgCl2, pH 6.8). p-nitrophenyl-α-d-glucopyranoside was used as substrate for the reaction and it was also dissolved in potassium phosphate buffer at 6 mmol/L. For the assay reaction, firstly 282 μL of Mangifera indica L. leaves extract and 200 μL substrate were mixed and incubated at 37 °C for 5 min and then 200 μL of enzyme solution was added. The enzyme reaction was carried out at 37 °C for 15 min, the reaction was stopped by 1.2 mL of glycine buffer (pH 10). Finally, enzyme activity was quantified spectrophotometrically by measuring absorbance at 410 nm. In order to measure inhibitory effect, the activity was compared with a control using water instead of MLE. The results were expressed as the sample concentration required to inhibit 50% of the enzyme activity (IC50). The IC50 values were graphically determined as the half-maximal inhibitory concentration of the inhibitor species giving 50% inhibition. Each assay was performed in triplicate.

### 4.7. Differentiation of 3T3-L1 into Adipocytes

3T3-L1 cells line were purchased from the ATCC (ATCC, Manassas, VA, USA). After thawing, 3T3-L1 cells were resuspended in a Dulbecco’s modified eagle’s medium (DMEM) 4.5 g/L d-glucose (Gibco by life technologies, Milan, Italy) supplemented with 10% *v*/*v* heat-inactivated fetal bovine serum (FBS; Gibco by life technologies, Milan, Italy), 1% penicillin/streptomycin (Carlo Erba, Milan, Italy) antibiotic/antimycotic solution and 60 mg/mL of gentamicin (Gibco by life technologies, Milan, Italy). The cultures were maintained at 37 °C in a 5% CO_2_ incubator, and medium was changed every 3 days. Subsequently, 3T3-L1 cells were sub-cultured in three groups:

Undifferentiated (UNDIFF), grown with Dulbecco’s modified eagle’s medium (DMEM) abovementioned; differentiated (DIFF), grown with adipogenic medium consisted of Dulbecco’s modified eagle’s medium (DMEM) 4.5 g/L d-glucose supplemented with 10% *v*/*v* of FBS, 10mg/mL insulin (Sigma-Aldrich, St. Louis, MO, USA), 0.5 mM dexamethasone (Sigma-Aldrich), and 0.1 mM indomethacin (Sigma-Aldrich); and Differentiated with MLE, grown with co-treatment of adipogenic medium and MLE concentration of 35 μg. Medium and treatment were changed every two days. Five days of differentiation later, cells were collected by trypsinization, washed once with PBS (phosphate-buffered saline), and then lysed for RNA extraction.

### 4.8. Oil Red Staining

Staining solution was achieved using 0.21% oil red O in 100% isopropanol (Sigma-Aldrich, St. Louis, MO, USA). Briefly, adipocytes, grown in 24-well plate, were fixed in 10% formaldehyde, stained with oil red O for 10 min, and washed with 60% isopropanol (Sigma-Aldrich), after the oil red O was eluted by adding 100% isopropanol, shacked gently for 10 min and then the optical density (OD) read at 490 nm for 0.5 s.

Lipid droplet size were acquired by analyzing the whole bottom surface area of an individual well of a 24-well plate. The pictures were obtained by using inverted multichannel LED fluorescence microscope (Evos, Life Technologies, Grand Island, NY, USA), as representative images of the experimental conditions. Each experiment was performed in triplicate.

### 4.9. RNA Extraction and qRT-PCR

Trizol reagent (Life Technology, Milan, Italy) was used to extract total RNA. It was subsequently converted into cDNA through an Applied Biosystem (Foster City, CA, USA) reverse transcription reagent. The quantitative analysis was performed with One-Step Fast Real-Time PCR System Applied Biosystem using the SYBR Green PCR master mix (Life Technology, Milan, Italy). The primer sequences used are shown in Table 2. The mix for PCR analyses included previously synthesized cDNA, SYBR green PCR master mix (Life Technology, Milan, Italy), primer mix (forward primer/reverse primer) and UltraPureTM Distilled Water DNase/RNase Free (Invitrogen by Life Technologies, Milan, Italy). PCR reactions were subjected to 40 cycles of 95 °C for 20 s, 95 °C for 3 s and 60 °C for 30 s. The relative mRNA expression levels of each gene were determined by the threshold cycle (*C*t) value of each PCR product and normalized with GAPDH (Glyceraldeyde-3-phosphate dehydrogenase) by using comparative 2^−∆∆*C*t^ method. The analysis was performed in duplicates.

### 4.10. GSH Determination

Levels of non-proteic thiol groups (RSH), about 90% of GSH content, were measured in 200 μL of cell lysate using a spectrophotometric assay based on the reaction of thiol groups with 2,2-dithio-bis-nitrobenzoic acid (DTNB) at *λ* = 412 nm (*εM* = 13,600).

### 4.11. Determination of Adiponectin by Enzyme-Linked Immunosorbent Assay (ELISA)

Cells were seeded at a constant density to obtain identical experimental conditions in the different tests and to achieve a high accuracy of the measurements. In order to determine Adiponectin protein content, 20 μL of cell lysate and culture supernatant respectively, were assayed by enzyme-linked immunosorbent assay (ELISA) (EMD Millipore Corporation, Billerica, MA, USA), according to the manufacturer’s guidelines. Results are expressed as ng/mg and ng/mL protein ± standard deviation (SD).

### 4.12. Statistical Analysis

Statistical significance (*p* < 0.05) of the differences between the experimental groups was determined by the Fisher method for the analysis of multiple comparisons. For the comparisons between the treatment groups, the null hypothesis was tested through single-factor analysis of variance (ANOVA) for multiple groups or the unpaired *t*-test for two groups. Data were presented as mean ± SD.

## 5. Conclusions

Taken together, data show that MLE from *Mangifera indica* might be used to create a new potential natural pharmacological strategy to counteract metabolic diseases such as obesity, diabetes and metabolic syndrome.

## Figures and Tables

**Table 1:** Methodology of evaluations and biological samples preparations are reported in a previous work (ng/mL ± standard deviation, *r*^2^, limit of detection (LOD) and LOQ) [18].

Compounds	Concentration (ng/mL)	LOD	LOQ	*r* ^2^
Mangiferin	70,200 ±150	1875	7320	0.9985
Myo-inositol	21,600 ± 230	832	4250	0.9973
γ-Orizanolo	47,700 ± 190	1432	5265	0.9987

**Table 2:** Primers used in this study.

Gene GenBank Code	Forward and Reverse Primers Sequence (5′→3′)	Tm °C	Amplicon Size
**Adiponectin** NM_009605	GAAGCCGCTTATGTGTATCGC	61.5	76
	GAATGGGTACATTGGGAACAGT	60.0	
**CAT** NM_009804.2	AAGATTGCCTTCTCCGGGTG	60.04	430
	TGTGGAGAATCGAACGGCAA	59.97	
**DGAT1** NM_010046.3	GTTTCCGTCCAGGGTGGTAG	287	871
	GTTGGATCAGCCCCACTTGA	1119	
**DGAT2** NM_026384.3	CAGGTGCCGTCTTGGGTTAT	1932	100
	CAGGAGGATATGCGCCAGAG	1993	
**FAS** NM_007988	GGAGGTGGTGATAGCCGGTAT	62.9	140
	TGGGTAATCCATAGAGCCCAG	60.4	
**GAPDH** NM_008085	AGCTTCGGCACATATTTCATCTG	61.0	89
	CGTTCACTCCCATGACAAACA	60.5	
**HMOX1** NM_010442.2	CCTCACAGATGGCGTCACTT	840	200
	TGGGGGCCAGTATTGCATTT	1001	
**Il-10** NM_010548	GCTGGACAACATACTGCTAACC	60.9	78
	ATTTCCGATAAGGCTTGGCAA	60.0	
**Il-6** NM_031168.2	CCCCAATTTCCAATGCTCTCC	598	141
	CGCACTAGGTTTGCCGAGTA	699	
**MnSOD** NM_009127.4	GCCCAAACCTATCGTGTCCA	3102	70
	AGGGAACCCTAAATGCTGCC	3133	
**PPARα** NM_011144.6	CCGAACATTGGTGTTCGCAG	135	161
	AGATACGCCCAAATGCACCA	257	
**PPARγ** 19016	TCGCTGATGCACTGCCTATG	62.4	103
	GAGAGGTCCACAGAGCTGATT	60.9	
**SCD1** NM_009127.4	GAGTAGCTGAGCTTTGGGCT	1378	591
	ACTTCATCAGCGGGGACTTG	1930	

## Abbreviations

AMPK	Adenosine monophosphate-activated protein kinase
CAT	Catalase
DGAT-1	Diacylglycerol-O-acyltransferase-1
DGAT-2	Diacylglycerol-O-acyltransferase-2
DMSO	Dimethyl sulfoxide
DPPH	1,1-Diphenyl-2-picryl-hydrazyl
FAS	Fatty Acid Synthase
FFA	Free fatty acids
GAPDH	Glyceraldeyde-3-phosphate dehydrogenase
GLUT4	Glucose transporter 4
GSH	Glutathione
HO-1	Heme oxygenase-1
IL-10	Interleukin-10
IL-6	Interleukin 6
LOD	Limit of detection
LOQ	Limit of quantification
MLE	*Mangifera indica* L. leaf extract
MnSOD	Manganese-dependent superoxide dismutase
OD	Optical density
ORZ	γ-oryzanol
PPAR-α	Peroxisome proliferator activated receptor-alpha
PPAR-γ	Peroxisome proliferator activated receptor-gamma
SCD-1	Stearoyl-coenzyme A desaturase 1
TG	Triglycerides
